# Migration and psychosis: a meta-analysis of incidence studies

**DOI:** 10.1017/S0033291719000035

**Published:** 2019-02-06

**Authors:** Jean-Paul Selten, Els van der Ven, Fabian Termorshuizen

**Affiliations:** 1 School for Mental Health and Neuroscience, University of Maastricht, Maastricht, The Netherlands; 2 GGZ Rivierduinen, Institute for Mental Health, Leiden, The Netherlands

**Keywords:** Bipolar disorder, depressive disorder, dopamine, ethnicity, migration, psychosis, race, schizophrenia, social exclusion, stress

## Abstract

**Background:**

The aims of this meta-analysis are (i) to estimate the pooled relative risk (RR) of developing non-affective psychotic disorder (NAPD) and affective psychotic disorder (APD) among migrants and their children; (ii) to adjust these results for socioeconomic status (SES); (iii) to examine the sources of heterogeneity that underlie the risk of NAPD.

**Methods:**

We included population-based incidence studies that reported an age-adjusted RR with 95% confidence interval (CI) published 1 January 1977–12 October 2017 and used a random-effects model.

**Results:**

We retrieved studies performed in Europe (*n* = 43), Israel (*n* = 3), Canada (*n* = 2) and Australia (*n* = 1). The meta-analysis yielded a RR, adjusted for age and sex, of 2.13 (95% CI 1.99–2.27) for NAPD and 2.94 (95% CI 2.28–3.79) for APD. The RRs diminished, but persisted after adjustment for SES. With reference to NAPD: a personal or parental history of migration to Europe from countries outside Europe was associated with a higher RR (RR = 2.94, 95% CI 2.63–3.29) than migration within Europe (RR = 1.88, 95% 1.62–2.18). The corresponding RR was lower in Israel (RR = 1.22; 0.99–1.50) and Canada (RR = 1.21; 0.85–1.74). The RR was highest among individuals with a black skin colour (RR = 4.19, 95% CI 3.42–5.14). The evidence of a difference in risk between first and second generation was insufficient.

**Conclusions:**

Positive selection may explain the low risk in Canada, while the change from exclusion to inclusion may do the same in Israel. Given the high risks among migrants from developing countries in Europe, social exclusion may have a pathogenic role.

## Introduction

Migrants face the difficult task of settling into the society of a new country. It is not surprising, therefore, that a recent meta-analysis found migrants to be at increased risk of developing mood disorders (pooled relative risk, RR, 1.25, 95% CI 1.11–1.41) (Mindlis and Boffetta, 2017). Meta-analytic evidence, however, suggests that migrants and their children are at an even higher risk for schizophrenia or other non-affective psychotic disorders (NAPDs), with RRs exceeding 2.0 (Cantor-Graae and Selten, 2005; Bourque *et al*., 2011).

As the last meta-analysis included studies up to 2008, there is a need for an up-to-date, comprehensive meta-analysis that estimates not only the risk of NAPD, but also that of affective psychotic disorder (APD), and that adjusts for socioeconomic status (SES) in the country of destination. Two competing theories have been proposed to explain the low SES of individuals who develop a psychotic disorder (PD): social causation (stress) and social selection (downward mobility of the genetically predisposed). Since there is little evidence of an association between parental SES and risk of psychosis, the mechanism of social selection may be more important than that of social causation (Dohrenwend *et al*., 1992; Kwok, 2014). Indeed, research has shown that a large part of this downward mobility occurs before the development of psychosis, in that many patients with psychosis fail to reach their expected educational level (Kendler *et al*., 2016). However, since a role for social causation cannot be entirely excluded, it is important to adjust any effect of migration for SES.

A previous meta-analysis reported that the risk of NAPD did not differ significantly between first- and second-generation migrants (Bourque *et al*., 2011), which suggests that ethnic minority status rather than migration is an important factor in the development of psychosis. In order to investigate this and other potential factors, we performed a meta-analysis of incidence studies (i) to estimate the pooled RR (*v.* the reference population) of developing APD, NAPD, or any of these disorders, among international migrants and their children; (ii) to adjust these results for SES; and (iii) to examine sources of heterogeneity with reference to the RR of NAPD, such as generational status (first or second), region of destination, region of origin, developmental level of country of origin, skin colour, refugee status and sex.

## Method

### Study selection

In order to be considered for the meta-analysis, studies were required (i) to report a risk ratio (RR, incidence rate ratio, hazard ratio or odds ratio) with 95% confidence interval (CI) for the incidence or prevalence of APD and/or NAPD among migrants in a circumscribed geographical area (or to provide numerators and denominators for the calculation of such measures); (ii) to adjust the risk ratio for differences in age between migrants and the reference population (or to provide data that make this adjustment possible); and (iii) to have been published in a peer-reviewed journal. For the purpose of the present study, we used incidence studies only.

For details of the study selection, see online Supplementary Methods and Supplementary Fig. S1.

### Quality check

Two authors (FT and EV) evaluated the quality of the articles independently, using the criteria given in online Supplementary Table S1 (range: 0–15). The inter-rater reliability of this procedure was good (intraclass correlation coefficient = 0.85). The averages of these scores were divided into tertiles and considered as indicating low (⩽9), medium (>9 to ⩽11) or high (>11) quality.

### Data extraction

Two authors (FT and JPS) extracted the effect sizes independently. They recorded information about diagnosis (NAPD, APD or PD without distinction between affective or non-affective), country of destination, country or region of origin, developmental level of country of origin, skin colour, refugee status, sex, birthplace (first or second generation), adjustment for age, sex and/or SES. In case of any discrepancy, consensus was reached by discussion.

### Meta-analysis

First, we calculated the pooled RR for the development of any PD (NAPD, APD or PD), NAPD and APD among migrants and their children worldwide, adjusted for age and sex (analysis 1.1 for any PD, 1.2 for NAPD, 1.3 for APD).

The analyses were performed using the ‘metan’ procedure of STATA (Palmer and Sterne, 2016). Given the significant heterogeneity across studies, the use of a random-effects model was indicated. This model assumes that there is heterogeneity between studies that is not due to within-study variance (i.e. the standard error of the effect estimate) and leads to downplaying of outliers and often a broader 95% CI.

We repeated the analyses using the STATA command ‘robumeta’, which takes into account clustering of effect estimates that originate from the same study, and compared the results to those obtained using ‘metan’.

The effect of study quality on inter-study heterogeneity was investigated using the ‘metareg’ procedure of STATA (analysis 2). Funnel plots were used to investigate possible publication bias (analysis 3).

Since only two studies adjusted the results for SES at birth (Hjern *et al*., 2004 for youth study group; Corcoran *et al*., 2009), we adjusted the results for current SES, i.e. at first contact for treatment of psychosis. For this purpose, we selected studies that reported two effect sizes: one adjusted for age and sex, and another one adjusted for age, sex and current SES. We then compared the two summary RRs (analysis 4.1 for any PD, 4.2 for NAPD).

The analyses below concern the incidence of NAPD only. We calculated the differences between subgroups in univariable models using ‘metareg’ (Palmer and Sterne, 2016). This was done in two ways: (a) using all available effect estimates; (b) using effect estimates for subgroups derived from the same study, to assess confounding by study (as with SES).

In analysis 5.1, we examined the difference in risk between first-generation and second-generation (born in country of destination to a foreign-born father and/or foreign-born mother) migrants. Average effect estimates were calculated for migrants and their children according to region of destination (analysis 5.2) and region of origin (analysis 5.3). Owing to the small number of studies presenting effect sizes for migrants from different regions, a comparison of effect sizes in the same study (as for SES) was not feasible here. In analysis 5.4, we compared the effect of migration to Europe from countries outside Europe to that of (international) migration within Europe.

We classified the developmental level of the country or region of origin according to the definitions used by the United Nations Conference on Trade and Development (United Nations, 2002) and compared the risk of PDs in migrants and their children from developed countries to that of their peers from developing countries (analysis 5.5).

The effect of skin colour was examined by comparing migrants and their children from areas where the majority of the population is white, black or other. Since most individuals from North Africa and the Middle East are considered white, but are visibly different from white Europeans, we created the following categories: (1) white, i.e. from Europe, North America or Australia; (2) white other (i.e. from North Africa or the Middle East, including Turkey); (3) black (i.e. from the Caribbean or sub-Saharan Africa); (4) other (e.g. India, China, Greenland) or mixed (e.g. South America, Africa); and (5) unknown (analysis 5.6). For the comparison of groups with different skin colours, we selected studies with at least three of these groups.

Analysis 5.7 investigated the effect of refugee status, and analysis 5.8 that of sex (migrants and their children). Lastly, the results of analyses 5.5 (developmental level of country of origin) and 5.6 (skin colour) were adjusted for SES in analyses 6.1 and 6.2.

## Results

### Result of computerized search

Forty-nine articles were retrieved, which concerned 43 independent observational studies or data sources. Table 1 lists the studies, performed in Europe (*n* = 37), Israel (*n* = 3), Canada (*n* = 2) or Australia (*n* = 1). Forty-seven articles concerned the risk of NAPD and four the risk of APD. At least 20 633 first- and second-generation migrants developed a PD (affective or non-affective) during 35 890 528 person-years at risk. At least 96 850 members of the reference population did so during 623 587 721 person-years. From six studies, the relevant numbers of cases and/or denominators could not be derived (Rwegellera, 1977; Mortensen *et al*., 1997; Fearon *et al*., 2006; Werbeloff *et al*., 2012; Bansal *et al*., 2014; Manhica *et al*., 2016).

**Table 1.** Population-based incidence studies included in meta-analysis of risk for psychosis associated with personal or parental history of migration, 1977–2017, by pertinent region of study, number of cases (migrants and non-migrants), diagnosis, study quality and type of analysis

Study	Country/region	Number of Cases^a^	Diagnosis	Quality	Included in analysis
Rwegellera (1977)	UK, London	35/47	NAPD^b^	Low	1.1 1.2 5.1 5.2 5.3 5.4 5.5 5.6 5.7
Hitch and Clegg (1980)	UK, Bradford	63/123	NAPD	Low	1.1 1.2 5.1 5.2 5.3 5.4 5.5 5.6 5.7 5.8
Krupinski (1980)	Australia,Victoria	423/1097	NAPD	Low	1.1 1.2 5.1 5.2 5.3 5.4 5.5 5.6 5.7 5.8
Dean *et al*. (1981)	UK, London	403/1191	NAPD	Low	1.1 1.2 5.1 5.2 5.3 5.4 5.5 5.6 5.7 5.8
McGovern and Cope (1987)	UK, Birmingham	51/98	NAPD	Low	1.1 1.2 5.2 5.3 5.4 5.5 5.6 5.7 5.8
Cochrane and Bal (1987)	UK	315/3669	NAPD	Low	1.1 1.2 5.1 5.2 5.3 5.4 5.5 5.6 5.7 5.8
Harrison *et al*. (1988)	UK, Nottingham	27/59	NAPD	Medium	1.1 1.2 5.1 5.2 5.3 5.4 5.5 5.6 5.7
Castle *et al*. (1991)	UK, London	36/53	NAPD	Medium	1.1 1.2 5.2 5.3 5.4 5.5 5.6 5.7
Thomas *et al*. (1993)	UK, Manchester	18/41	NAPD	Low	1.1 1.2 5.1 5.2 5.3 5.4 5.5 5.6 5.7 5.8
Selten and Sijben (1994)	The Netherlands	166/975	NAPD	Low	1.1 1.2 5.1 5.2 5.3 5.4 5.5 5.6 5.7 5.8
van Os *et al*. (1996)	UK, London	61/44	NAPD	Medium	1.1 1.2 5.2 5.3 5.4 5.5 5.6 5.7
Selten *et al*. (1997)	The Netherlands	933/10 726	NAPD	Medium	1.1 1.2 5.1 5.2 5.3 5.4 5.5 5.6 5.7 5.8
Harrison *et al*. (1997)	UK, Nottingham	32/136	NAPD	High	1.1 1.2 5.2 5.3 5.4 5.5 5.6 5.7 5.8
Bhugra *et al*. (1997)	UK, London	62/76	NAPD	High	1.1 1.2 5.2 5.3 5.4 5.5 5.6 5.7 5.8
Mortensen *et al*. (1997)	Denmark	725/?	NAPD	Low	1.1 1.2 5.1 5.2 5.7 5.8
Goater *et al*. (1999)	UK, London	44/29	NAPD	High	1.1 1.2 5.2 5.3 5.4 5.5 5.6 5.7
Selten *et al*. (2001)	NL, The Hague	116/65	PD^c^	High	1.1 4.1 4.2 6.5 6.6
Zolkowska *et al*. (2001)	Sweden, Malmö	22/34	NAPD	High	1.1 1.2 5.1 5.2 5.7
Reeves *et al*. (2001)	UK, London	36/25	NAPD	Medium	1.1 1.2 5.1 5.2 5.3 5.4 5.5 5.6 5.7 5.8
Cantor-Graae *et al*. (2003)	Denmark	1023/9221	NAPD	Medium	1.1 1.2 5.1 5.2 5.3 5.4 5.5 5.6 5.7
Mitter *et al*. (2004)	UK, London	13/27	NAPD	High	1.1 1.2 5.1 5.2 5.3 5.4 5.5 5.6 5.7 5.8
Hjern *et al*. (2004)	Sweden	249/1339	NAPD	Low	1.1 1.2 5.1 5.2 5.3 5.4 5.5 5.6 5.7
Cantor-Graae *et al*. (2005)	Sweden, Malmö	15/56	NAPD	High	1.1 1.2 5.1 5.2 5.7
Fearon *et al*. (2006)	UK, London	568 (all)	PD/NAPD/ APD^d^	High	1.1 1.2 1.3 5.2 5.3 5.4 5.5 5.6 5.7 5.8
Leao *et al*. (2006)	Sweden	754/1305	NAPD	Medium	1.1 1.2 4.1 4.2 5.1 5.2 5.3 5.4 5.5 5.6 ***5.7*** 5.8 6.5 6.6
Veling *et al*. (2006)	NL, The Hague	300/79	NAPD	High	1.2 5.1 5.2 5.3 5.4 5.5 5.6 5.7 5.8
Smith *et al*. (2006)	Canada, Br. Col.	548/259	NAPD	Medium	1.1 1.2 5.1 5.2 5.3 5.4 5.5 5.6 5.7
Weiser *et al*. (2008)	Israel	1640/46	NAPD	Medium	1.1 1.2 4.1 4.2 5.1 5.2 5.3 5.4 5.5 5.6 5.7 6.5 6.6
Cantor-Graae and Pedersen (2007*a*)	Denmark	905/9742	NAPD	Medium	1.1 1.2 5.2 5.2 5.3 5.4 5.5 5.6 5.7
Cantor-Graae and Pedersen (2007*b*)	Denmark	112/4472	NAPD	Medium	1.1 1.2 5.1 5.2 5.3 5.4 5.5 5.6 5.7 5.8
Coid *et al*. (2008)^§^	UK, London	280/82	NAPD	High	5.1
Kirkbride *et al*. (2008)^§^	UK, London	372/112	PD/NAPD/APD	High	1.1 1.2 1.3 4.1 4.2 4.3 5.2 5.3 5.4 5.5 5.6 5.7 5.8 6.5 6.6
Corcoran *et al*. (2009)	Israel	445/192	NAPD	Low	1.1 1.2 5.1 5.2 5.7
Zandi *et al*. (2010)	NL, Utrecht	15/32	PD/NAPD	High	1.1 1.2 5.2 5.3 5.4 5.5 5.6 5.7 5.8
Cheng *et al*. (2011)	UK, Cambridge	66/206	PD	High	1.1
Werbeloff *et al*. (2012)	Israel	2335/?	NAPD	Low	1.1 1.2 5.1 5.2 5.3 5.4 5.5 5.6 5.7 5.8
Tarricone *et al*. (2012)	Italy, Bologna	35/128	PD/NAPD	High	1.1 1.2 5.1 5.2 5.7
Sendra-Gutierrez *et al*. (2012)	Spain, Segovia	9/59	NAPD	Medium	1.1 1.2 5.1 5.2 5.7
Tortelli *et al*., (2014)	France, Paris	122/ 136	PD	Low	1.1
Cantor-Graae and Pedersen (2013)	Denmark	3665/16 203	NAPD	High	1.1 1.2 5.1 5.2 5.7
Lasalvia *et al*. (2014)	Italy, Veneto	127/431	PD/NAPD/APD	High	1.1 1.2 1.3 5.2 5.7
Bansal *et al*. (2014)	Scotland	815/6861	NAPD	Low	1.1 1.2 4.1 4.2 5.2 5.3 5.4 5.5 5.6 5.7 5.8 6.5 6.6
Anderson *et al*. (2015)	Canada, Ontario	2305/20 959	NAPD	Medium	1.1 1.2 5.1 5.2 5.3 5.4 5.5 5.6 ***5.7***
Hollander *et al*. (2016)	Sweden	472/3232	NAPD	Medium	1.1 1.2 4.1 4.2 5.1 5.2 5.3 5.4 5.5 5.6 ***5.7*** 5.8 6.5 6.6
Manhica *et al*. (2016)	Sweden	?/?	NAPD	Medium	1.1 1.2 4.1 4.2 5.1 5.2 5.3 5.4 5.5 5.6 ***5.7*** 6.5 6.6
Mule *et al*. (2017)	Italy, Palermo	21/183	PD/NAPD	High	1.1 1.2 5.1 5.2 5.7
Kirkbride *et al*. (2017*a*) Kirkbride *et al*. (2017*b*)^¶^	UK, East Anglia	173/514	PD/NAPD/APD	High	1.1 1.2 1.3 4.1 4.2 4.3 5.1 5.2 5.3 5.4 5.5 5.6 5.7 6.5 6.6
Barghadouch *et al*. (2016)	Denmark	95/297	NAPD	High	4.1 4.2 6.5 6.6
Markkula *et al*. (2017)	Finland	1494/2388	NAPD	High	4.1 4.2 6.5 6.6

a Among migrants/among natives.

b Non-affective psychotic disorder.

c Psychotic disorder, affective or non-affective.

d Affective psychotic disorder.

***5.7***, estimates for refugees, 5.7 estimates for migrants and/or their children, irrespective of refugee status.

§Two papers about the same study.

¶Two papers about the same study.

### Meta-analysis

**Fig. 1.**
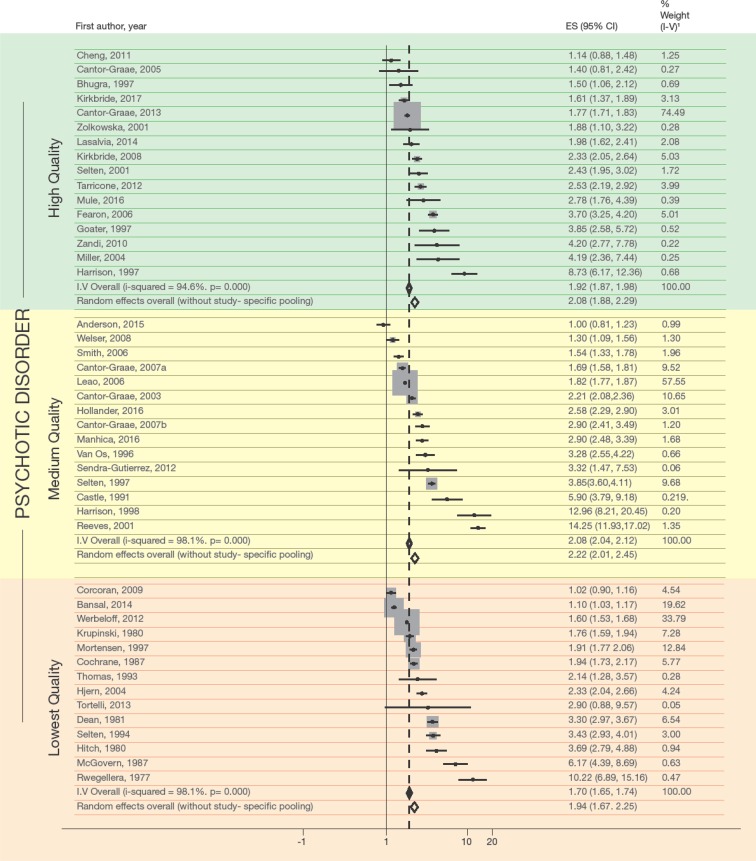
Meta-analysis of incidence studies examining the association between migration and psychosis, 1977–2017. Effect estimates for any psychotic disorder (i.e. psychotic disorder or non-affective psychotic disorder), by study quality. When a study reported separate effect sizes for PD and for NAPD, that for PD was selected, because it concerned the largest number of cases.

Low-quality studies yielded somewhat lower, but not significantly different, effect measures for any PD or NAPD than medium- or high-quality studies (Table 2; Fig. 1). The risk of developing APD was somewhat higher than the risk of developing NAPD. As expected, there was significant heterogeneity across studies.

**Table 2.** Meta-analysis of incidence studies examining the association between a personal or parental history of migration and psychosis, 1977–2017

Type of psychotic disorder	*N*^a^	RR^b^	95% CI	*I*^2^ (%)^c^	Δ lnRR ^d^	*p*	RR	95% CI	*I*^2^ (%)
Any psychotic disorder	45^e^	2.10	1.97–2.24	98.1					
Low quality	14	1.94	1.67–2.25	98.1	ref				
Medium quality	15	2.22	2.01–2.45	98.8	0.148	0.186			
High quality	16	2.08	1.88–2.29	94.6	0.076	0.519			
Adjusted for age, sex, SES (using all estimates available)	10^f^						1.53	1.41–1.67	95.2
Adjusted for age and sex (paired observations)	8^g^						1.72	1.57–1.87	98.0
Adjusted for age, sex, SES (paired observations)	8^g^						1.56	1.43–1.70	92.5
Non-affective psychotic disorder	43^h^	2.13	1.99–2.27	98.1					
Low quality	13	1.94	1.67–2.25	98.3	ref				
Medium quality	15	2.22	2.01–2.45	98.8	0.152	0.172			
High quality	15	2.15	1.95–2.37	94.7	0.124	0.297			
Adjusted for age, sex, SES (using all estimates available)	10^f^						1.55	1.42–1.69	95.3
Adjusted for age and sex (paired observations)	8^g^						1.74	1.59–1.90	98.0
Adjusted for age, sex, SES (paired observations)	8^g^						1.57	1.44–1.71	92.9
Affective psychotic disorder	4	2.94	2.28–3.79	69.1					
Low quality	0								
Medium quality	0								
High quality	4	2.94	2.28–3.79	69.1					

Pooled relative risks, by psychosis type and quality of study. Idem, analysis restricted to incidence studies that adjusted the results for age and sex, as well as for age, sex and socioeconomic status (SES).

a Number of papers.

b Pooled relative risk, adjusted for age and sex.

c Measure of heterogeneity. All values were statistically significant.

d The difference between the logarithmically transformed RR of psychotic disorder among migrants *v*. natives in a certain category and the logarithmically transformed RR in the reference category.

e The number does not equal 49, because two papers (Barghadouch *et al*., 2016; Markkula *et al*., 2017) reported effect sizes adjusted for age, sex and SES (no effect size unadjusted for SES). From overlapping papers, Selten *et al.* (2001) was selected instead of Veling *et al*. (2006) and Kirkbride *et al*. (2008) instead of Coid *et al*. (2008).

f Eight studies that reported an effect size adjusted for age and sex, as well as an effect size adjusted for age, sex and SES (Selten, 2001; Leao *et al*., 2006; Weiser *et al*., 2008; Kirkbride *et al*., 2008; Bansal *et al*., 2014; Hollander *et al*., 2016; Manhica *et al*., 2016; Kirkbride *et al*., 2017*a*, 2017*b*) plus two studies that reported an effect size adjusted for age, sex and SES (Barghadouch *et al*., 2016; Markkula *et al*., 2017).

g Derived from the above eight studies that reported an effect size adjusted for age and sex, and an effect size adjusted for age, sex and SES.

h The number does not equal 46, because two papers (Barghadouch *et al*., 2016; Markkula *et al*., 2017) reported effect sizes adjusted for age, sex and SES (no effect size unadjusted for SES). From overlapping papers, Veling *et al*. (2006) was selected instead of Selten *et al.* (2001).

The RRs estimated using the STATA command ‘robumeta’ were a little higher and had somewhat broader 95% CIs, but remained statistically significant (results available on request).

The funnel plot of studies of the risk of any PD showed evidence of publication bias (online Supplementary Fig. S1; Egger's test *p* = 0.01). We therefore performed a sensitivity analysis by excluding studies with standard errors >0.2 and by omitting a study that reported an excessive RR, >10.0 (Harrison *et al*., 1988). The pooled RR of any PD decreased from RR 2.10 (95% CI 1.97–2.24) to RR 1.92 (95% CI 1.81–2.05) and Egger's test was no longer significant (*p* = 0.098). When we also excluded the low-quality studies, the RR of any PD was 1.97 (95% CI 1.84–2.10). Using robumeta, this figure was 2.23 (95% CI 1.80–2.76).

Application of these analyses to studies of the risk of NAPD yielded similar results: when we excluded the studies with standard errors >2.0 and the Harrison *et al*. study, the RR was 1.93 (1.82–2.06). Using robumeta, the RR was 2.18 (1.86–2.55). When we also excluded the low-quality studies, the RR of NAPD was 1.98 (1.86–2.12), with robumeta: 2.19 (1.82–2.64).

Eight studies adjusted findings for SES. Although adjustment for SES resulted in a decrease in the RR of any PD (from RR 1.72, 95% CI 1.57–1.87, to RR 1.56, 95% CI 1.43–1.70), this measure remained significant. The same was true when the RR of APD and NAPD was adjusted for SES (Table 2).

When we considered all the available effect sizes with reference to NAPD, the RR of psychosis among first-generation migrants was significantly higher than that among second-generation migrants. However, when we restricted the analyses to those publications that reported effect sizes for both generations, the difference in RR became smaller and statistically not significant (Table 3).

**Table 3.** Meta-analysis of incidence studies examining the association between migration and psychosis, 1977–2017

	Any estimate available						Estimates derived from the same study					
Subgroups	*N*^a^	RR of NAPD^b^	95% CI	*I*^2^ (%)^c^	Δ lnRR^d^	*p*	*N*	RR of NAPD	95% CI	*I*^2^ (%)	Δ lnRR	*p*
Generational status												
First generation	29	2.55	2.31–2.82	97.9	ref		9	2.05	1.85–2.27	89.9	ref	
Second generation	13	1.78	1.66–1.90	94.2	−0.35	<0.001	9	1.80	1.68–1.93	81.6	−0.11	0.196
Developmental level of country of origin												
Developed country^e^	19	1.66	1.49–1.84	97.1	ref		15	1.43	1.25–1.65	90.7	ref	
Developing country^e^	30	2.54	2.26–2.86	97.6	0.43	<0.001	15	2.13	1.89–2.41	89.7	0.39	<0.001
Skin colour^f^												
White	19	1.65	1.46–1.85	97.1	ref		7	1.24	0.96–1.59	81.8	ref	
White other	8	1.94	1.54–2.44	87.6	0.15	0.334	3	1.86	1.23–1.81	83.9	0.40	0.095
Black	23	4.19	3.42–5.14	94.3	0.94	<0.001	7	2.78	2.12–3.63	86.3	0.80	<0.001
Other	11	1.73	1.41–2.14	95.1	0.05	0.685	5	1.52	1.12–2.05	90.5	0.20	0.288
Unknown/mixed	32	1.83	1.71–1.96	96.5	0.11	0.194	5	1.53	1.18–1.99	68.2	0.21	0.30
Refugee status												
Refugee	4	1.88	1.57–2.24	91.4	−0.15	0.342	4	1.87	1.56–2.24	91.5	0.066	0.524
Refugee or non-refugee	43	2.15	2.01–2.31	98.1	ref							
Non-refugee							4	1.75	1.59–1.93	89.5	ref	
Sex												
Male	22	2.25	1.99–2.54	98.3	ref		21	2.24	1.98–2.54	95.6	ref	
Female	21	2.26	2.01–2.53	97.2	0.011	0.915	21	2.26	2.01–2.53	92.5	0.012	0.912

Analysis of variables that may moderate the association between a personal or parental history of migration and incidence of non-affective psychotic disorder.

a Number of papers.

b Pooled relative risk of non-affective psychotic disorder, adjusted for age and sex. (except the analysis for sex differences: adjusted for age only).

c Measure of heterogeneity. All values were statistically significant.

d Difference between the logarithmically transformed RR of non-affective psychotic disorder among migrants *v*. natives in a certain category and the logarithmically transformed RR in the reference category.

e According to UNCTAD definition. United Nations: UNCTAD Handbook of Statistics. Geneva, United Nations Conference on Trade and Development, 2002.

f Predominant skin colour in region of origin. ‘White other’ refers to individuals from North-Africa or the Middle East. The results depicted in the right column are derived from a comparison of at least three effect sizes from the same study: one for a white group, a second one for a black group and a third one for another subgroup (white other, other or unknown/mixed).

The results presented below apply to migrants *and* their children, except one analysis on refugees.

The RR of developing NAPD was lowest in Israel and Canada and significantly higher in all European destinations (Table 4). Region of origin had a substantial impact on this RR, which was highest among first- and second-generation migrants from Central and South America (mostly from the Caribbean), sub-Saharan countries and North Africa, and lowest among migrants from the Indian subcontinent or other parts of Asia (Table 4).

**Table 4.** Meta-analysis of incidence studies examining the association between a personal or parental history of migration and psychosis, 1977–2017

Destination	Number of papers	RR of NAPD	95% CI	*I*^2^ (%)^a^
Great-Britain	18	2.69	2.20–3.28	98.7
Scandinavia	11	1.89	1.79–2.00	93.0
The Netherlands	4	2.98	2.43–3.66	84.5
Southern Europe	4	2.79	1.94–4.01	83.6
Israel	3	1.22	0.99–1.50	95.5
Canada	2	1.21	0.85–1.74	91.2
Australia	1	2.10	1.22–3.82	n.a.
Region of origin				
Eastern Europe	7	1.93	1.38–2.70	93.3
Western Europe or ‘Europe’	13	1.62	1.41–1.86	98.0
Indian subcontinent	7	1.65	1.31–2.07	79.6
Remainder of Asia or ‘Asia’	15	1.52	1.22–1.89	89.2
North-Africa	4	2.88	1.85–4.49	68.7
Sub-Saharan Africa or ‘Africa’	13	2.99	2.30–3.89	91.6
Central- or South-America	23	3.01	2.53–3.58	93.3
Other or unknown	28	1.89	1.76–2.04	97.1
Combination of region of origin and destination				
From outside Europe to Europe	10	2.94	2.63–3.29	95.7
Within Europe	28	1.88	1.62–2.18	98.3

Influence of destination and region of origin on risk of non-affective psychotic disorder (NAPD).

a Measure of heterogeneity. All values were statistically significant.

In Europe, the RR was higher for migrants and their children who came from countries outside Europe (RR 2.94, 95% CI 2.63–3.29) than among their peers from within Europe (RR 1.88, 95% CI 1.62–2.18). The funnel plot for the first analysis (from outside Europe to Europe) showed no evidence of publication bias (online Supplementary Fig. S1; Egger's test *p* = 0.24).

The developmental level of the country of origin had a substantial impact: the RR of NAPD was higher among migrants from developing countries (Table 3). However, a black skin was the variable with the largest impact on the risk of NAPD (Table 3). Black migrants and their children were not only at a higher risk than members of the reference population in Europe, but also in Canada (pooled effect size for migrants from the Caribbean and Bermuda, West, East and Central Africa, RR 1.55, 95% CI 1.13–1.87) (Anderson *et al*., 2015) and Israel (migrants from Ethiopia, hazard ratio 3.02, 95% CI 1.93–4.73) (Weiser *et al*., 2008). The results showed no significant difference in RR between those with a white or other non-black skin colour.

The risk of NAPD was higher among refugees than among natives, but not significantly higher than that among migrants and their children in general. Four studies compared foreign-born refugees to foreign-born non-refugees, but the pooled RRs were not significantly different (Table 3).

The effect of sex was negligible (Table 3).

After adjustment for SES, the RR of NAPD among first- and second-generation migrants from developing countries and among those with a black skin was still increased (RR 1.84, 95% CI 1.55–2.19, and RR 2.70, 95% CI 1.99–3.68, respectively) (online Supplementary Table S2).

Owing to the small numbers of studies, it was not possible to repeat all the analyses using ‘robumeta’. However, where this was possible, the conclusions remained the same.

## Discussion

Although findings indicate that migrants are at increased risk of developing APD and NAPD, the findings were heterogeneous, which precludes a conclusion about ‘the’ RR among migrants in general. While there was no evidence of a greatly increased risk among migrants in Israel or Canada, the risk among migrants in Europe, in particular those from developing countries outside Europe and/or those with a black skin, was greatly increased. Overall, the risk became somewhat attenuated after adjustment for SES at first contact. A comparison of risks for first- and second-generation migrants based on effect estimates derived from the same study yielded no significant difference. This indicates that membership of a disadvantaged ethnic minority group, rather than a personal history of migration, is an important determinant of risk. There was insufficient evidence to conclude that there is a difference in risk between refugees and non-refugees.

It is unlikely that the findings can be explained by bias. Some researchers (e.g. Zandi *et al*., 2010) have argued that Western psychiatrists misunderstand patients from a different culture, but most studies of clinical presentation and long-term outcome do not indicate that there is a major diagnostic bias (e.g. Morgan *et al*., 2017).

Furthermore, this meta-analysis showed that the risk of PDs was high among migrants from Eastern Europe, who do not differ greatly in culture from Western Europeans. For a thorough discussion of these issues, see previous reviews (Cantor-Graae and Selten, 2005; Bourque *et al*., 2011). Selection bias is an unlikely explanation, because field studies of major mental disorders have shown that the ratio of treated to untreated cases is negatively associated with SES (Link and Dohrenwend, 1980). There was evidence of publication bias, but its effect was modest.

Strengths of this meta-analysis are the large number of studies included, and, with reference to Europe, the diversity of the migrant groups examined. By using a ‘pairwise’ comparison of effect sizes derived from the same study (e.g. effect sizes adjusted and non-adjusted for SES), it was often possible to avoid confounding by study.

Our meta-analysis has several limitations. First, our results apply mainly to Europe. Of note, studies of first hospital admissions published before 1977 reported a modestly increased risk of schizophrenia among migrants to the USA. Malzberg (1964), for example, reported that the first admission rates among foreign-born white individuals in New York City, standardized for age and sex, exceeded those among the native-born white individuals by 16%. To our knowledge, no investigation has reported a greatly increased risk among migrants to the USA or Canada (Selten and Cantor-Graae, 2004).

Second, the quality of information on the risk for refugees was limited. Only one study compared the risk for refugees to non-refugees from the same part of the world (Hollander *et al*., 2016). The result of this investigation, a significantly higher risk for refugees, remains inconclusive, however, because the parts of the world were as large as Asia. Since Asia comprises countries as diverse as Afghanistan and China, the comparability of refugees and non-refugees remains uncertain. Leao *et al*. (2006) designated migrants from particular countries (e.g. African countries) refugees, while the authors did not know whether these subjects were the victims of political persecution. Consequently, the outcome of our meta-analysis, i.e. insufficient evidence of a difference in risk between refugees and non-refugees, should not be interpreted as evidence of no difference.

Third, the adjustment for current, rather than parental SES. This is problematic because low SES at onset of psychosis may also be the result of a disturbed neuro-development. Of note, there is a larger potential for downward mobility during the pre-psychotic period among natives than among migrants or their children, many of whom already belong to a lower social class. The situation is complex, because many migrants may have belonged to a higher social class before migration. Also, the definitions of SES varied considerably.

Fourth, the lower rates for migrants within Europe might be explained to some extent by the fact that it is relatively easy to seek support in the country of origin.

Finally, the arbitrary and imperfect classification of subjects on the basis of their skin colour. One could argue, for instance, that subjects from the Indian subcontinent are often darker than Africans and that the group ‘other’, consisting of people from South-America and the larger part of Asia is very heterogeneous. However, given the relatively small number of studies, we opted for this classification. With the designation of a group as white (e.g. Eastern Europeans), we do not suggest that this group is not exposed to prejudice or discrimination.

How should the findings be interpreted? It was previously thought that individuals with a genetic predisposition for psychosis are more likely to migrate than others (Odegaard, 1932), but not a single study has supported this negative selection hypothesis (Cantor-Graae and Selten, 2005). On the contrary, many studies from the USA and Canada have reported a better (somatic) health among migrants than among native-born individuals, while the results of similar studies from Europe vary greatly across migrant populations (Ikram *et al*., 2016). In any event, due to the self-selection of healthy, resilient people who have the courage to move to a new environment, migrants from developing countries are probably healthier than those who stay behind. Current knowledge about the years before the first hospital admission, during which the patient often exhibits a lack of initiative (Hafner *et al*., 1999), provides additional evidence against the negative selection hypothesis. Receiving countries can reinforce a positive selection process through their immigrant admission policies. Since such policies are more rigorous in Canada and Australia than in Europe, the relatively low risk of PDs among many migrant groups in the first two countries may be due to positive selection (Vang *et al*., 2017). Lastly, selective migration cannot explain the increased risk among Ethiopian Jews, because the whole population moved to Israel (Spector, 2005).

There is no evidence that the high incidence of psychosis among migrants from developing countries in Europe reflects a similarly high incidence in the country of origin. While there have been no high-quality incidence studies from Africa, studies from the Caribbean (Bhugra *et al*., 1996), Surinam (Selten *et al*., 2005), India and China (Baxter *et al*., 2016) have reported ‘normal’ incidence or prevalence rates. This also implies that poverty by itself is an unlikely cause of psychosis.

However, as many migrants who move from a developing country to Europe find themselves in the lowest strata of European society, the effect of migration might be due, at least in part, to a (relative) social disadvantage (Morgan *et al*., 2008) or social defeat. Indeed, a case–control study from the UK found strong associations between indicators of social disadvantage and psychosis. Indicators of disadvantage and isolation were more common in Black Caribbean subjects than in White British subjects (Morgan *et al*., 2008).

The social defeat hypothesis of psychosis, which proposes that the negative experience of being excluded from the majority group increases the risk of psychosis by sensitizing the mesolimbic dopamine system, postulates a link with the brain (Selten and Cantor-Graae, 2005; Selten *et al*., 2016). Sensitization of the mesolimbic dopamine system refers to an increased dopamine function in response to stressors and becomes manifest in excessive presynaptic dopamine synthesis and release (Howes and Murray, 2014). Several lines of evidence support the social defeat hypothesis: (i) the risk of psychosis is increased in other groups exposed to social exclusion, such as individuals with a history of trauma or bullying in childhood, homosexuals, African-Americans, individuals with a low IQ or hearing impairment (for review, see Selten *et al*., 2017); (ii) the protective effect of high ethnic density, i.e. residence in a neighbourhood where the own ethnic group is well-represented (Schofield *et al*., 2017); (iii) experiments with rodents that demonstrate dopamine sensitization in defeated animals (Hammels *et al*., 2015); (iv) a recent positron emission tomography study showing increased dopamine synthesis and increased stress-induced dopamine release in the striatum of individuals (healthy volunteers, clinical high-risk subjects and schizophrenia patients) with a personal or parental history of migration (Egerton *et al*., 2017). Neuro-receptor imaging studies of non-psychotic individuals with a history of hearing impairment or childhood trauma have also reported dopamine sensitization (Gevonden *et al*., 2014; Oswald *et al*., 2014; Egerton *et al*., 2016). Lastly, the pattern of findings in Israel (i.e. a modest increase in risk among first-generation non-black migrants and the absence of an increase in risk among second-generation migrants) may fit with this interpretation, because the migration of Jews to a Jewish state involves a change from social exclusion to inclusion.

An entirely different interpretation of the increased risk among individuals with a dark skin is that low prenatal levels of vitamin D are a risk factor for psychosis (McGrath, 1999). Indeed, a study from the Netherlands reported that pregnant women and infants from non-European ethnic backgrounds are at high risk of vitamin D deficiency (Vinkhuyzen *et al*., 2016). However, the evidence of a causal role for low vitamin D in the aetiology of psychosis is inconclusive and the hypothesis does not explain the increased risk among first-generation migrants.

Given the fact that substance abuse in the general population is a lot more common among men than among women, the absence of a difference in RR between male and female migrants argues against a major role of drug abuse in the aetiology of the increased incidence.

There is anecdotal evidence and a tiny body of research to suggest that the ratio of risk among migrants and natives is inversed in countries where the original population occupies the weaker position. This may apply, for example, to Aboriginals in Australia, Maori in New Zealand, Native Americans in the USA and Inuit in Canada (Sampath, 1974; Tapsell *et al*., 2018). This is an important topic for further study.

In conclusion, the results of this meta-analysis confirm earlier European findings of an increased incidence of NAPD among migrants from developing countries, in particular those of African extraction (Cantor-Graae and Selten, 2005; Bourque *et al*., 2011), and extend them by showing that individuals with an African background are also at an increased risk of PD in Canada and Israel, while migration to the latter countries is generally not associated with an increased risk.

From the large number of research implications, we would like to point out the importance of examining the interaction between ethnic background and host country: does the risk for an immigrant group vary according to the country of destination and which are the determinants of this? A prospective study could test the social defeat hypothesis by comparing striatal dopamine synthesis capacity between migrants and natives in early adult age at two points in time: shortly after arrival in the country of destination and 3–5 years later. The hypothesis predicts a greater rise in striatal dopamine synthesis capacity among migrants than among natives (or a rise in this capacity among migrants and no such rise among natives).

We conclude that the increased psychosis risk among migrants and their children is a major public health problem. Since the lifetime morbid risk for psychosis (affective or non-affective) is about 1–2% for Europeans, our findings suggest that this risk may be 3–6% for migrants from outside Europe. The challenge is to advance our understanding of the underlying mechanisms and to find ways for prevention.

## Supplementary material

For supplementary material accompanying this paper visit https://doi.org/10.1017/S0033291719000035. click here to view supplementary material

## References

[ref2] Anderson KK, Cheng J, Susser E, McKenzie KJ and Kurdyak P (2015) Incidence of psychotic disorders among first-generation immigrants and refugees in Ontario. Canadian Medical Association Journal 187, E279–E286. 25964387 10.1503/cmaj.141420 PMC4467956

[ref3] Bansal N, Bhopal R, Netto G, Lyons D, Steiner MF and Sashidharan SP (2014) Disparate patterns of hospitalisation reflect unmet needs and persistent ethnic inequalities in mental health care: the Scottish health and ethnicity linkage study. Ethnicity and Health 19, 217–239. 23844602 10.1080/13557858.2013.814764

[ref4] Barghadouch A, Carlsson J and Norredam M (2016) Psychiatric disorders and predictors hereof among refugee children in early adulthood: a register-based cohort study. Journal of Nervous and Mental Disease 206, 3–10. 10.1097/NMD.0000000000000576 27483113

[ref5] Baxter AJ, Charlson FJ, Cheng HG, Shidhaye R, Ferrari AJ and Whiteford HA (2016) Prevalence of mental, neurological, and substance use disorders in China and India: a systematic analysis. The Lancet. Psychiatry 3, 832–841. 27528097 10.1016/S2215-0366(16)30139-0

[ref6] Bhugra D, Hilwig M, Hossein B, Marceau H, Neehall J, Leff J, Mallett R and Der G (1996) First-contact incidence rates of schizophrenia in Trinidad and one-year follow-up. British Journal of Psychiatry 169, 587–592. 8932887 10.1192/bjp.169.5.587

[ref7] Bhugra D, Leff J, Mallett R, Der G, Corridan B and Rudge S (1997) Incidence and outcome of schizophrenia in whites, African-Caribbeans and Asians in London. Psychological Medicine 27, 791–798. 9234457 10.1017/s0033291797005369

[ref8] Bourque F, van der Ven E and Malla A (2011) A meta-analysis of the risk for psychotic disorders among first- and second-generation immigrants. Psychological Medicine 41, 897–910. 20663257 10.1017/S0033291710001406

[ref14] Cantor-Graae E and Pedersen CB (2007a) Risk for schizophrenia in intercountry adoptees: a Danish population-based cohort study. Journal of Child Psychology Psychiatry 48, 1053–1060. 18030684 10.1111/j.1469-7610.2007.01788.x

[ref15] Cantor-Graae E and Pedersen CB (2007b) Risk of schizophrenia in second-generation immigrants: a Danish population-based cohort study. Psychological Medicine 37, 485–494. 17202000 10.1017/S0033291706009652

[ref16] Cantor-Graae E and Pedersen CB (2013) Full spectrum of psychiatric disorders related to foreign migration: a Danish population-based cohort study. JAMA Psychiatry 70, 427–435. 23446644 10.1001/jamapsychiatry.2013.441

[ref17] Cantor-Graae E and Selten JP (2005) Schizophrenia and migration: a meta-analysis and review. American Journal of Psychiatry 162, 12–24. 15625195 10.1176/appi.ajp.162.1.12

[ref18] Cantor-Graae E, Pedersen CB, McNeil TF and Mortensen PB (2003) Migration as a risk factor for schizophrenia: a Danish population-based cohort study. British Journal of Psychiatry 182, 117–122. 12562738 10.1192/bjp.182.2.117

[ref19] Cantor-Graae E, Zolkowska K and McNeil TF (2005) Increased risk of psychotic disorder among immigrants in Malmo: a 3-year first-contact study. Psychological Medicine 35, 1155–1163. 16116941 10.1017/s0033291705004721

[ref21] Castle D, Wessely S, Der G and Murray RM (1991) The incidence of operationally defined schizophrenia in Camberwell, 1965–84. British Journal of Psychiatry 159, 790–794. 1790446 10.1192/bjp.159.6.790

[ref22] Cheng F, Kirkbride JB, Lennox BR, Perez J, Masson K, Lawrence K, Hill K, Feeley L, Painter M, Murray GK, Gallagher O, Bullmore ET and Jones PB (2011) Administrative incidence of psychosis assessed in an early intervention service in England: first epidemiological evidence from a diverse, rural and urban setting. Psychological Medicine 41, 949–958. 21205440 10.1017/S0033291710002461

[ref24] Cochrane R and Bal SS (1987) Migration and schizophrenia: an examination of five hypotheses. Social Psychiatry 22, 181–191. 3686161 10.1007/BF00583553

[ref25] Coid JW, Kirkbride JB, Barker D, Cowden F, Stamps R, Yang M and Jones PB (2008) Raised incidence rates of all psychoses among migrant groups: findings from the East London first episode psychosis study. Archives of General Psychiatry 65, 1250–1258. 18981336 10.1001/archpsyc.65.11.1250

[ref26] Corcoran C, Perrin M, Harlap S, Deutsch L, Fennig S, Manor O, Nahon D, Kimhy D, Malaspina D and Susser E (2009) Incidence of schizophrenia among second-generation immigrants in the Jerusalem perinatal cohort. Schizophrenia Bulletin 35, 596–602. 18648022 10.1093/schbul/sbn089 PMC2669576

[ref28] Dean G, Walsh D, Downing H and Shelley E (1981) First admissions of native-born and immigrants to psychiatric hospitals in South-East England 1976. British Journal of Psychiatry 139, 506–512. 7332854 10.1192/bjp.139.6.506

[ref29] Dohrenwend BP, Levav I, Shrout PE, Schwartz S, Naveh G, Link BG, Skodol AE and Stueve A (1992) Socioeconomic status and psychiatric disorders: the causation-selection issue. Science 255, 946–952. 1546291 10.1126/science.1546291

[ref30] Egerton A, Valmaggia LR, Howes OD, Day F, Chaddock CA, Allen P, Winton-Brown TT, Bloomfield MAP, Bhattacharyya S, Chilcott J, Lappin JM, Murray RM and McGuire P (2016) Adversity in childhood linked to elevated striatal dopamine function in adulthood. Schizophrenia Research 176, 171–176. 27344984 10.1016/j.schres.2016.06.005 PMC5147458

[ref31] Egerton A, Howes OD, Houle S, McKenzie K, Valmaggia LR, Bagby MR, Tseng HH, Bloomfield MA, Kenk M, Bhattacharyya S, Suridjan I, Chaddock CA, Winton-Brown TT, Allen P, Rusjan P, Remington G, Meyer-Lindenberg A, McGuire PK and Mizrahi R (2017) Elevated striatal dopamine function in immigrants and their children: a risk mechanism for psychosis. Schizophrenia Bulletin 43, 293–301. 28057720 10.1093/schbul/sbw181 PMC5605255

[ref32] Fearon P, Kirkbride JB, Morgan C, Dazzan P, Morgan K, Lloyd T, Hutchinson G, Tarrant J, Fung WL, Holloway J, Mallett R, Harrison G, Leff J, Jones PB, Murray RM and Group AS (2006) Incidence of schizophrenia and other psychoses in ethnic minority groups: results from the MRC AESOP Study. Psychological Medicine 36, 1541–1550. 16938150 10.1017/S0033291706008774

[ref34] Gevonden M, Booij J, van den Brink W, Heijtel D, van Os J and Selten JP (2014) Increased release of dopamine in the striata of young adults with hearing impairment and its relevance for the social defeat hypothesis of schizophrenia. JAMA Psychiatry 71, 1364–1372. 25271822 10.1001/jamapsychiatry.2014.1325

[ref35] Goater N, King M, Cole E, Leavey G, Johnson-Sabine E, Blizard R and Hoar A (1999) Ethnicity and outcome of psychosis. British Journal of Psychiatry 175, 34–42. 10621766 10.1192/bjp.175.1.34

[ref36] Hafner H, Loffler W, Maurer K, Hambrecht M and an der Heiden W (1999) Depression, negative symptoms, social stagnation and social decline in the early course of schizophrenia. Acta Psychiatrica Scandinavica 100, 105–118. 10480196 10.1111/j.1600-0447.1999.tb10831.x

[ref37] Hammels C, Pishva E, De Vry J, van den Hove DL, Prickaerts J, van Winkel R, Selten JP, Lesch KP, Daskalakis NP, Steinbusch HW, van Os J, Kenis G and Rutten BP (2015) Defeat stress in rodents: from behavior to molecules. Neuroscience and Biobehavoral Reviews 59, 111–140. 10.1016/j.neubiorev.2015.10.006 26475995

[ref38] Harrison G, Owens D, Holton A, Neilson D and Boot D (1988) A prospective study of severe mental disorder in Afro-Caribbean patients. Psychological Medicine 18, 643–657. 3263659 10.1017/s0033291700008321

[ref39] Harrison G, Glazebrook C, Brewin J, Cantwell R, Dalkin T, Fox R, Jones P and Medley I (1997) Increased incidence of psychotic disorders in migrants from the Caribbean to the United Kingdom. Psychological Medicine 27, 799–806. 9234458 10.1017/s0033291796004643

[ref40] Hitch PJ and Clegg P (1980) Modes of referral of overseas immigrant and native-born first admissions to psychiatric hospital. Social Science & Medicine 14A, 369–374. 10.1016/0160-7979(80)90120-4 7394580

[ref41] Hjern A, Wicks S and Dalman C (2004) Social adversity contributes to high morbidity in psychoses in immigrants – a national cohort study in two generations of Swedish residents. Psychological Medicine 34, 1025–1033. 15554573 10.1017/s003329170300148x

[ref43] Hollander AC, Dal H, Lewis G, Magnusson C, Kirkbride JB and Dalman C (2016) Refugee migration and risk of schizophrenia and other non-affective psychoses: cohort study of 1.3 million people in Sweden. BMJ 352, i1030. 26979256 10.1136/bmj.i1030 PMC4793153

[ref44] Howes OD and Murray RM (2014) Schizophrenia: an integrated sociodevelopmental-cognitive model. Lancet 383, 1677–1687. 24315522 10.1016/S0140-6736(13)62036-X PMC4127444

[ref45] Ikram UZ, Mackenbach JP, Harding S, Rey G, Bhopal RS, Regidor E, Rosato M, Juel K, Stronks K and Kunst AE (2016) All-cause and cause-specific mortality of different migrant populations in Europe. European Journal of Epidemiology 31, 655–665. 26362812 10.1007/s10654-015-0083-9 PMC4977342

[ref46] Kendler KS, Ohlsson H, Mezuk B, Sundquist JO and Sundquist K (2016) Observed cognitive performance and deviation from familial cognitive aptitude at age 16 years and ages 18 to 20 years and risk for schizophrenia and bipolar illness in a Swedish National Sample. JAMA Psychiatry 73, 465–471. 27028264 10.1001/jamapsychiatry.2016.0053

[ref48] Kirkbride JB, Barker D, Cowden F, Stamps R, Yang M, Jones PB and Coid JW (2008) Psychoses, ethnicity and socio-economic status. British Journal of Psychiatry 193, 18–24. 18700213 10.1192/bjp.bp.107.041566

[ref49] Kirkbride JB, Hameed Y, Ankireddypalli G, Ioannidis K, Crane CM, Nasir M, Kabacs N, Metastasio A, Jenkins O, Espandian A, Spyridi S, Ralevic D, Siddabattuni S, Walden B, Adeoye A, Perez J and Jones PB (2017 *a*) The epidemiology of first-episode psychosis in early intervention in psychosis services: findings from the social epidemiology of psychoses in East Anglia [SEPEA] study. American Journal of Psychiatry 174, 143–153. 27771972 10.1176/appi.ajp.2016.16010103 PMC5939990

[ref50] Kirkbride JB, Hameed Y, Ioannidis K, Ankireddypalli G, Crane CM, Nasir M, Kabacs N, Metastasio A, Jenkins O, Espandian A, Spyridi S, Ralevic D, Siddabattuni S, Walden B, Adeoye A, Perez J and Jones PB (2017b) Ethnic minority status, age-at-immigration and psychosis risk in rural environments: evidence from the SEPEA study. Schizophrenia Bulletin 43, 1251–1261. 28521056 10.1093/schbul/sbx010 PMC5737276

[ref52] Krupinski JC (1980) Migration and mental health – a comparative study. Journal of Intercultural Studies 1, 49–57.

[ref53] Kwok W (2014) Is there evidence that social class at birth increases risk of psychosis? a systematic review. International Journal of Social Psychiatry 60, 801–808. 24608029 10.1177/0020764014524737

[ref54] Lasalvia A, Bonetto C, Tosato S, Zanatta G, Cristofalo D, Salazzari D, Lazzarotto L, Bertani M, Bissoli S, De Santi K, Cremonese C, De Rossi M, Gardellin F, Ramon L, Zucchetto M, Amaddeo F, Tansella M, Ruggeri M and Group PI-V (2014) First-contact incidence of psychosis in north-eastern Italy: influence of age, gender, immigration and socioeconomic deprivation. British Journal of Psychiatry 205, 127–134. 24723631 10.1192/bjp.bp.113.134445

[ref55] Leao TS, Sundquist J, Johansson LM, Johansson SE and Sundquist K (2005) Incidence of mental disorders in second-generation immigrants in Sweden: a four-year cohort study. Ethnicity and Health 10, 243–256. 16087456 10.1080/13557850500096878

[ref56] Leao TS, Sundquist J, Frank G, Johansson LM, Johansson SE and Sundquist K (2006) Incidence of schizophrenia or other psychoses in first- and second-generation immigrants: a national cohort study. Journal of Nervous and Mental Disease 194, 27–33. 16462552 10.1097/01.nmd.0000195312.81334.81

[ref57] Link B and Dohrenwend BP (1980) Formulation of hypotheses about the ratio of untreated to treated cases in the true prevalence studies of functional psychiatric disorders in adults in the United States In Dohrenwend BP, Dohrenwend BS, Gould MS, Link B, Neugebauer R and Wunsch-Hirzig R (eds), Mental Illness in the United States: Epidemiological Estimates. New York: Praeger, pp. 133–148.

[ref58] Malzberg B (1964) Mental disease among native and foreign-born whites in New York state, 1949–1951. Mental Hygiene 48, 478–499. 14172429

[ref59] Manhica H, Hollander AC, Almquist YB, Rostila M and Hjern A (2016) Origin and schizophrenia in young refugees and inter-country adoptees from Latin America and East Africa in Sweden: a comparative study. British Journal of Psychiatry Open 2, 6–9. 27703747 10.1192/bjpo.bp.115.002048 PMC4998945

[ref60] Markkula N, Lehti V, Gissler M and Suvisaari J (2017) Incidence and prevalence of mental disorders among immigrants and native Finns: a register-based study. Social Psychiatry and Psychiatric Epidemiology 52, 1523–1540. 28856385 10.1007/s00127-017-1432-7

[ref61] McGovern D and Cope RV (1987) First psychiatric admission rates of first and second generation Afro Caribbeans. Social Psychiatry 22, 139–149. 3498221 10.1007/BF00583848

[ref62] McGrath J (1999) Hypothesis: is low prenatal vitamin D a risk-modifying factor for schizophrenia? Schizophrenia Research 40, 173–177. 10638855 10.1016/s0920-9964(99)00052-3

[ref65] Mindlis I and Boffetta P (2017) Mood disorders in first- and second-generation immigrants: systematic review and meta-analysis. British Journal of Psychiatry 210, 182–189. 28069564 10.1192/bjp.bp.116.181107

[ref66] Mitter PR, Krishnan S, Bell P, Stewart R and Howard RJ (2004) The effect of ethnicity and gender on first-contact rates for schizophrenia-like psychosis in Bangladeshi, Black and White elders in Tower Hamlets, London. International Journal of Geriatric Psychiatry 19, 286–290. 15027045 10.1002/gps.1084

[ref68] Morgan C, Kirkbride J, Hutchinson G, Craig T, Morgan K, Dazzan P, Boydell J, Doody GA, Jones PB, Murray RM, Leff J and Fearon P (2008) Cumulative social disadvantage, ethnicity and first-episode psychosis: a case-control study. Psychological Medicine 38, 1701–1715. 19000327 10.1017/S0033291708004534

[ref69] Morgan C, Fearon P, Lappin J, Heslin M, Donoghue K, Lomas B, Reininghaus U, Onyejiaka A, Croudace T, Jones PB, Murray RM, Doody GA and Dazzan P (2017) Ethnicity and long-term course and outcome of psychotic disorders in a UK sample: the AESOP-10 study. British Journal of Psychiatry 211, 88–94. 28642258 10.1192/bjp.bp.116.193342 PMC5537567

[ref70] Mortensen PB, Cantor-Graae E and McNeil TF (1997) Increased rates of schizophrenia among immigrants: some methodological concerns raised by Danish findings. Psychological Medicine 27, 813–820. 9234460 10.1017/s0033291797004741

[ref71] Mule A, Sideli L, Capuccio V, Fearon P, Ferraro L, Kirkbride JB, La Cascia C, Sartorio C, Seminerio F, Tripoli G, Di Forti M, La Barbera D and Murray RM (2017) Low incidence of psychosis in Italy: confirmation from the first epidemiological study in Sicily. Social Psychiatry and Psychiatric Epidemiology 52, 155–162. 28032136 10.1007/s00127-016-1322-4 PMC5333812

[ref75] Odegaard O (1932) Emigration and insanity. Acta Psychiatrica Neurologica Scandinavica 4, 1–206.

[ref76] Oswald LM, Wand GS, Kuwabara H, Wong DF, Zhu S and Brasic JR (2014) History of childhood adversity is positively associated with ventral striatal dopamine responses to amphetamine. Psychopharmacology *(*Berlin*)* 231, 2417–2433. 24448898 10.1007/s00213-013-3407-z PMC4040334

[ref77] Palmer TM and Sterne JAC (2016) Meta-Analysis in STATA: An Updated Collection From Stata Journal. Texas: Stata.

[ref79] Reeves SJ, Sauer J, Stewart R, Granger A and Howard RJ (2001) Increased first-contact rates for very-late-onset schizophrenia-like psychosis in African- and Caribbean-born elders. Britidsh Journal of Psychiatry 179, 172–174. 10.1192/bjp.179.2.172 11483481

[ref80] Rwegellera GG (1977) Psychiatric morbidity among West Africans and West Indians living in London. Psychologial Medicine 7, 317–329. 10.1017/s0033291700029421 877196

[ref81] Sampath HM (1974) Prevalence of psychiatric disorders in a southern Baffin Island Eskimo settlement. Canadian Psychiatric Association J 19, 363–367. 10.1177/070674377401900406 4426018

[ref82] Schofield P, Thygesen M, Das-Munshi J, Becares L, Cantor-Graae E, Pedersen C and Agerbo E (2017) Ethnic density, urbanicity and psychosis risk for migrant groups – a population cohort study. Schizophrenia Research 190, 82–87. 28318842 10.1016/j.schres.2017.03.032 PMC5735221

[ref84] Selten JP and Cantor-Graae E (2004) Schizophrenia and migration In Gattaz W and Hafner H (eds), Search for the Cause of Schizophrenia. Darmstadt, Germany: Steinkopff/Springer, pp. 3–25.

[ref85] Selten JP and Cantor-Graae E (2005) Social defeat: risk factor for schizophrenia? British Journal of Psychiatry 187, 101–102. 16055818 10.1192/bjp.187.2.101

[ref86] Selten JP and Sijben N (1994) First admission rates for schizophrenia in immigrants to The Netherlands. The Dutch National Register. Social Psychiatry and Psychiatric Epidemiology 29, 71–77. 8009322 10.1007/BF00805625

[ref88] Selten JP, Booij J, Buwalda B and Meyer-Lindenberg A (2017) Biological mechanisms whereby social exclusion may contribute to the etiology of psychosis: a narrative review. Schizophrenia Bulletin 42, 287–292. 10.1093/schbul/sbw180 PMC5782499 28053019

[ref89] Selten JP, Slaets JP and Kahn RS (1997) Schizophrenia in Surinamese and Dutch Antillean immigrants to The Netherlands: evidence of an increased incidence. Psychological Medicine 27, 807–811. 9234459 10.1017/s0033291797005199

[ref90] Selten JP, van Os J and Cantor-Graae E (2016) The social defeat hypothesis of schizophrenia: issues of measurement and reverse causality. World Psychiatry 15, 294–295. 27717278 10.1002/wps.20369 PMC5032495

[ref91] Selten JP, Veen N, Feller W, Blom JD, Schols D, Camoenie W, Oolders J, van der Velden M, Hoek HW, Rivero VM, van der Graaf Y and Kahn R (2001) Incidence of psychotic disorders in immigrant groups to The Netherlands. British Journal of Psychiatry 178, 367–372. 11282817 10.1192/bjp.178.4.367

[ref92] Selten JP, Zeyl C, Dwarkasing R, Lumsden V, Kahn RS and Van Harten PN (2005) First-contact incidence of schizophrenia in Surinam. British Journal of Psychiatry 186, 74–75. 15630127 10.1192/bjp.186.1.74

[ref93] Sendra-Gutierrez JM, de Francisco Beltran P, Iribarren M and Vargas Aragon ML (2012) [Outpatient psychiatric care in the immigrant population of Segovia (2001–2008): descriptive study]. Revista de Psiquiatria y Salud Mental 5, 173–182. 22854612 10.1016/j.rpsm.2011.05.004

[ref94] Smith GN, Boydell J, Murray RM, Flynn S, McKay K, Sherwood M and Honer WG (2006) The incidence of schizophrenia in European immigrants to Canada. Schizophrenia Research 87, 205–211. 16905294 10.1016/j.schres.2006.06.024

[ref95] Spector S (2005) Operation Solomon. The Daring Rescue of the Ethiopian Jews. New York: Oxford University Press.

[ref97] Tapsell R, Hallett C and Mellsop G (2018) The rate of mental health service use in New Zealand as analysed by ethnicity. Australasian Psychiatry 26, 290–293. 28691522 10.1177/1039856217715989

[ref98] Tarricone I, Mimmi S, Paparelli A, Rossi E, Mori E, Panigada S, Carchia G, Bandieri V, Michetti R, Minenna G, Boydell J, Morgan C and Berardi D (2012) First-episode psychosis at the West Bologna Community Mental Health Centre: results of an 8-year prospective study. Psychological Medicine 42, 2255–2264. 22394476 10.1017/S0033291712000335

[ref99] Thomas CS, Stone K, Osborn M, Thomas PF and Fisher M (1993) Psychiatric morbidity and compulsory admission among UK-born Europeans, Afro-Caribbeans and Asians in central Manchester. British Journal of Psychiatry 163, 91–99. 8353706 10.1192/bjp.163.1.91

[ref100] Tortelli A, Morgan C, Szoke A, Nascimento A, Skurnik N, de Caussade EM, Fain-Donabedian E, Fridja F, Henry M, Ezembe F and Murray RM (2014) Different rates of first admissions for psychosis in migrant groups in Paris. Social Psychiatry and Psychiatric Epidemiol 49, 1103–1109. 10.1007/s00127-013-0795-7 PMC4283097 24270936

[ref101] United Nations (2002) UNCTAD Handbook of Statistics. Geneva: United Nations Conference on Trade and Development.

[ref102] van Os J, Castle DJ, Takei N, Der G and Murray RM (1996) Psychotic illness in ethnic minorities: clarification from the 1991 census. Psychological Medicine 26, 203–208. 8643760 10.1017/s0033291700033845

[ref103] Vang ZM, Sigouin J, Flenon A and Gagnon A (2017) Are immigrants healthier than native-born Canadians? A systematic review of the healthy immigrant effect in Canada. Ethnicity and Health 22, 209–241. 27809589 10.1080/13557858.2016.1246518

[ref104] Veling W, Selten JP, Veen N, Laan W, Blom JD and Hoek HW (2006) Incidence of schizophrenia among ethnic minorities in the Netherlands: a four-year first-contact study. Schizophrenia Research 86, 189–193. 16839747 10.1016/j.schres.2006.06.010

[ref106] Vinkhuyzen AAE, Eyles DW, Burne TH, Blanken LME, Kruithof CJ, Verhulst F, Jaddoe VW, Tiemeier H and McGrath JJ (2016) Prevalence and predictors of vitamin D deficiency based on maternal mid-gestation and neonatal cord bloods: the generation R study. Journal of Steroid Biochemisrty and Molecular Biology 164, 161–167. 10.1016/j.jsbmb.2015.09.018 26385604

[ref107] Weiser M, Werbeloff N, Vishna T, Yoffe R, Lubin G, Shmushkevitch M and Davidson M (2008) Elaboration on immigration and risk for schizophrenia. Psychological Medicine 38, 1113–1119. 17988415 10.1017/S003329170700205X

[ref108] Werbeloff N, Levine SZ and Rabinowitz J (2012) Elaboration on the association between immigration and schizophrenia: a population-based national study disaggregating annual trends, country of origin and sex over 15 years. Social Psychiatry and Psychiatric Epidemiology 47, 303–311. 21286683 10.1007/s00127-011-0342-3

[ref111] Zandi T, Havenaar JM, Smits M, Limburg-Okken AG, van Es H, Cahn W, Algra A, Kahn RS and van den Brink W (2010) First contact incidence of psychotic disorders among native Dutch and Moroccan immigrants in the Netherlands: influence of diagnostic bias. Schizophrenia Research 119, 27–33. 20332065 10.1016/j.schres.2010.02.1059

[ref112] Zolkowska K, Cantor-Graae E and McNeil TF (2001) Increased rates of psychosis among immigrants to Sweden: is migration a risk factor for psychosis? Psychological Medicine 31, 669–678. 11352369 10.1017/s0033291701003786

