# Supplementary material for: Migration and psychosis: a meta-analysis of incidence studies
Source: Psychol Med. 2019 Feb 6;50(2):303–13. doi: 10.1017/S0033291719000035 (PMC7083571; doi:10.1017/S0033291719000035)
Supplement: Supplementary file 1 [file S0033291719000035sup001.zip › PsychMedLegendSuppplFig2.docx]

**Legend to Supplementary Figure 2.**

Funnel plot of studies examining the association between (A) a personal or parental history of migration and the relative risk (vs. non-migrants) of any psychotic disorder, 1977-2017; (B) a personal or parental history of migration from a country outside Europe and the relative risk (vs. non-migrants) of developing non-affective psychotic disorder in Europe, 1977-2017.

Each funnel plot shows:

1. the standard error (SE) against the logarithmically transformed RR (lnRR). In the absence of publication bias and over-dispersion (i.e., heterogeneity), the points should resemble a symmetrical inverted funnel.
2. Egger’s regression (*in red*) showing the SE plotted ln RR against the lnRR. Egger’s test for funnel plot A was significant (p=.01); for plot B non-significant (p= .24).
