# Supplementary material for: Migration and psychosis: a meta-analysis of incidence studies
Source: Psychol Med. 2019 Feb 6;50(2):303–13. doi: 10.1017/S0033291719000035 (PMC7083571; doi:10.1017/S0033291719000035)
Supplement: Supplementary file 1 [file S0033291719000035sup001.zip › PsychMedSupplTable1.docx]

**Supplementary Table 1. Description of six criteria, with corresponding categories and scores, for assessing the quality of incidence studies included in the meta-analysis that examines the association between a personal or parental history of migration and psychosis, 1977-2017.**

| **Quality criterium** | **Description** | **Categories** | **Score** |
| --- | --- | --- | --- |
| C1 | How were cases ascertained? | Inpatients and outpatients | 2 |
|  |  | Community survey or multiple institutions | 2 |
|  |  | Hospital admissions only | 1 |
|  |  | Unspecified | 0 |
| C2 | Was a leakage study conducted? | Yes | 1 |
|  |  | No | 0 |
| C3 | How were patients diagnosed? | Diagnostic system (e.g., DSM, ICD) | 2 |
|  |  | Clearly described, validated criteria | 1 |
|  |  | No system or not specified | 0 |
| C4 | Method of diagnostic assignment | Diagnostic interview (e.g., CASH) | 3 |
|  |  | Standardized case note review | 2 |
|  |  | Clinical diagnosis in notes or registries | 1 |
|  |  | Unspecified | 0 |
| C5 | Inter-rater reliability on diagnosis? | Yes | 1 |
|  |  | No | 0 |
| C6 | **Data quality** |  |  |
|  | Raw numerator reported | Yes | 1 |
|  |  | No | 0 |
|  | Raw denominator reported | Yes | 1 |
|  |  | No | 0 |
|  | Sex standardized | Yes | 1 |
|  |  | No | 0 |
|  | Age standardized in max 10 year age bands | Yes | 1 |
|  |  | No | 0 |
|  | Numerator and denominator match in time | Yes | 1 |
|  |  | No | 0 |
|  | Numerator and denominator match in space | Yes | 1 |
|  |  | No | 0 |
|  |  | Total score | Range 0-15 |

DSM=Diagnostic and Statistical Manual of Mental Disorders;

ICD=International Classification of Diseases;

CASH=Comprehensive Assessment of Symptoms and History (Andreasen, NC, Flaum M, Arndt S. The comprehensive assessment of symptoms and history (CASH): an instrument for assessing diagnosis and psychopathology. *Arch Gen Psychiatry*, 1992; 49: 615-623.
