# Supplementary material for: Migration and psychosis: a meta-analysis of incidence studies
Source: Psychol Med. 2019 Feb 6;50(2):303–13. doi: 10.1017/S0033291719000035 (PMC7083571; doi:10.1017/S0033291719000035)
Supplement: Supplementary file 1 [file S0033291719000035sup001.zip › PsychMedSupplTable2.docx]

**Supplementary Table 2. Meta-analysis of incidence studies examining the association between a personal or parental history of migration and psychosis, 1977-2017. Effect of developmental level of country of origin and skin colour on risk for non-affective psychotic disorder (NAPD), adjusted for age, sex and socio-economic status.**

|  | Any estimate available |  |  |  |  |  |  | Estimates derived from same study |  |  |  |  |  |
| --- | --- | --- | --- | --- | --- | --- | --- | --- | --- | --- | --- | --- | --- |
| **Subgroups** | Number of papers | RR of NAPD | 95% CI | I^2^ (%) ^1)^ | Delta  lnRR ^2)^ | p |  | Number of papers | RR of NAPD | 95% CI | I^2^ (%) ^1)^ | Delta  lnRR ^2)^ | p |
| **Developmental level of country of origin** |  |  |  |  |  |  |  |  |  |  |  |  |  |
| Developed country ^3)^ | 7 | 1.37 | 1.19-1.57 | 97.8 | ref |  |  | 6 | 1.01 | 0.89-1.14 | 58.8 | ref |  |
| Developing country ^3)^ | 9 | 2.00 | 1.88-2.13 | 93.0 | 0.30 | 0.003 |  | 6 | 1.66 | 1.35-2.05 | 85.0 | 0.505 | <.001 |
|  |  |  |  |  |  |  |  |  |  |  |  |  |  |
| **Skin colour** ^4)^ |  |  |  |  |  |  |  |  |  |  |  |  |  |
| White | 8 | 1.30 | 1.10-1.53 | 97.6 | ref |  |  | 5 | 1.02 | 0.85-1.21 | 67.8 | ref |  |
| White Other | 5 | 1.55 | 0.98-2.45 | 89.9 | 0.139 | 0.474 |  | 4 | 1.30 | 0.83-2.03 | 87.0 | 0.23 | 0.333 |
| Black | 8 | 2.70 | 1.99-3.68 | 90.9 | 0.725 | <0.001 |  | 5 | 2.55 | 1.79-3.64 | 87.4 | 0.91 | <0.001 |
| Other | 3 | 1.31 | 0.99-1.75 | 78.8 | 0.016 | 0.912 |  | 2 | 1.25 | 0.87-1.80 | 55.3 | 0.20 | 0.374 |
| Unknown/mixed | 10 | 1.43 | 1.30-1.57 | 87.6 | 0.089 | 0.341 |  | 5 | 1.19 | 0.95-1.49 | 47.1 | 0.16 | 0.393 |
|  |  |  |  |  |  |  |  |  |  |  |  |  |  |
|  |  |  |  |  |  |  |  |  |  |  |  |  |  |
|  |  |  |  |  |  |  |  |  |  |  |  |  |  |

1. Measure of heterogeneity. All values were statistically significant.
2. Difference between the logarithmically transformed RR of non-affective psychotic disorder among migrants vs. natives in a certain category and the logarithmically transformed RR in the reference category.
3. According to UNCTAD Definition. Reference: United Nations: UNCTAD Handbook of Statistics. Geneva, United Nations Conference on Trade and Development, 2002.
4. Predominant skin colour in region of origin. “White other” refers to individuals from North-Africa or the Middle East. The results depicted in the right column have been derived from a comparison of at least three effect sizes from the same study: one for a white group, a second one for a black group and a third one for another subgroup (white, other, other of mixed/unknown).
