# Supplementary material for: Migration and psychosis: a meta-analysis of incidence studies
Source: Psychol Med. 2019 Feb 6;50(2):303–13. doi: 10.1017/S0033291719000035 (PMC7083571; doi:10.1017/S0033291719000035)
Supplement: Supplementary file 1 [file S0033291719000035sup001.zip › S0033291719000035sup001.docx]

**Supplementary Methods (for online-Supplement)**

The last version of the study protocol was registered on 12 February 2018 with PROSPERO: CRD42017055488 ([www.crd.york.ac.uk.prospero](http://www.crd.york.ac.uk.prospero)).

Using the combination of keywords {“psychotic disorder” OR “schizophrenia” OR “psychosis” OR “mania” OR “affective”} AND {migrant OR migrants OR immigrant OR immigrants OR ethnic OR ethnicity} AND {ratio OR incidence OR odds}, and the special feature “explosion”, we searched the PubMed, Embase and PsychInfo databases for relevant articles in English, French or German published between 1 January 1977 and 12 October 2017. The lower limit of 1977 was selected, because a publication in that year (Cochrane, 1977) constituted a marked progress over many previous studies that failed to adjust the results for age differences between migrants and natives.

Three researchers (either FT & Olga Warmerdam [see Acknowledgement] or FT & EvdV) screened 8305 records, independently, and identified 69 articles suitable for in-depth evaluation. Fifty-two of 69 papers were already known to the authors. A further seven articles were identified by reading the reference lists of the obtained articles: four did not provide the keywords indicative of an epidemiological analysis (Hitch and Clegg, 1980; Krupinski, 1980; McGovern and Cope, 1987; Rwegellera, 1977), but were included in an earlier meta-analysis (Cantor-Graae and Selten, 2005), one study concerned intercountry adoptees (Cantor-Graae and Pedersen, 2007a), and two studies escaped the selection for other reasons (Hogerzeil *et al.*, 2017; Manhica *et al.*, 2016). Conference abstracts were excluded. A summary of the selection procedure and number of screened and selected articles is given in Supplementary Figure 1. We contacted the first authors of three papers for additional information (Bansal *et al.*, 2014; Kirkbride *et al.*, 2017a; Kirkbride *et al.*, 2017b).

Seven of the 76 selected articles were excluded for the following reasons: age correction was not performed (Boydell *et al.*, 2013; Carpenter and Brockington, 1980; Nielssen *et al.*, 2013), psychotic and non-psychotic disorders were included in the same effect size (Bruxner *et al.*, 1997), an ethnic minority population was included instead of a migrant group (Bresnahan *et al.*, 2007), or a deviant effect measure was used without population-based denominator data (Dealberto *et al.*, 2011; Kroll *et al.*, 2011). A further 11 studies were excluded because they investigated the prevalence of psychotic disorder (Amad *et al.*, 2013; Brugha *et al.*, 2004; Gabrielsen and Kramp, 2009; McGrath *et al.*, 2001; Menezes *et al.*, 2011; Norredam *et al.*, 2009, 2010; Qassem *et al.*, 2015; Schrier *et al.*, 2001; Selten *et al.*, 2012; Wijesinghe and Clancy, 1991).

The remaining 58 articles were checked for full or partial overlap in study population. Nine articles were excluded because of overlap with another article: King *et al*., 1994, because of overlap with Goater *et al*., 1999; Boydell *et al*., 2001, because of overlap with van Os *et al*., 1996; Mitter *et al*., 2005, because of overlap with Reeves *et al.,* 2001; Sundquist *et al*. 2004 because of overlap with Westman *et al*., 2006 and Leao *et al.*, 2006; Westman *et al*., 2006, because of overlap with Leao *et al.*, 2006; Leao *et al.*, 2005, because of overlap with Leao *et al*., 2006; Veling *et al.* 2011., because of overlap with Veling *et al*., 2006; Hogerzeil *et al*., 2017, because of overlap with Veling *et al*., 2011, and Veling *et al*., 2006; and Schofield *et al.* 2017, because of overlap with Cantor-Graae and Pedersen, 2013.

Six other pairs of articles overlapped in study population, but were retained because each article contributed in its own way to the present analysis. Some articles used a different definition of outcome (e.g., schizophrenia versus non-schizophrenic psychotic disorder) or a different classification or subpopulation of interest (e.g., first vs. second generation): Mortensen *et al*., 1997 (estimates for diagnostic category ‘NAPD other than Schizophrenia’) and Cantor-Graae *et al.*, 2003 (estimates for diagnostic category ‘Schizophrenia’); Selten *et al.,* 2001 (retained for analysis of psychotic disorder, without distinction between APD and NAPD) and Veling *et al*., 2006 (for the analysis of NAPD); Hjern *et al*., 2004 (patients younger than 20 years) and Leao *et al*., 2006 (patients aged 20 years and older); Cantor-Graae *et al.,* 2005 (estimates for second-generation migrants) and Hollander *et al.,* 2016 (estimates for first-generation migrants); Coid *et al*., 2008 (separate estimates for first- and second-generation migrants) and Kirkbride *et al*., 2008 (no distinction by generation); Hollander *et al*., 2016 and Manhica *et al*., 2016 (estimate for refugees from East-Africa in Manhica *et al*., 2016 excluded because of presence of the same subgroup in Hollander *et al.*, 2016).

Most studies based the designation of migrant status on the country of birth of the subjects in question or their parents. Several studies conducted in the UK, however, were based on categories derived from national censuses, with some studies using “whites” or “the remainder of the general population” as the reference group. Thus, some members of the reference group may actually have migrated to the UK , and some members of ethnic minority populations may have lived in the UK for more than two generations. This type of classification error would, however, primarily tend toward an attenuation of the relative risks currently obtained for migrants (Cantor-Graae and Selten, 2005). Thus, the present study designates the reference group as “the reference population”.

**References**

**Amad, A., Guardia, D., Salleron, J., Thomas, P., Roelandt, J. L. & Vaiva, G.** (2013). Increased prevalence of psychotic disorders among third-generation migrants: results from the French Mental Health in General Population survey. *Schizophrenia Research* **147**, 193-195.

**Bansal, N., Bhopal, R., Netto, G., Lyons, D., Steiner, M. F. & Sashidharan, S. P.** (2014). Disparate patterns of hospitalisation reflect unmet needs and persistent ethnic inequalities in mental health care: the Scottish health and ethnicity linkage study. *Ethnicity and Health* **19**, 217-39.

**Bhugra, D., Hilwig, M., Hossein, B., Marceau, H., Neehall, J., Leff, J., Mallett, R. & Der, G.** (1996). First-contact incidence rates of schizophrenia in Trinidad and one-year follow-up. *British Journal of Psychiatry* **169**, 587-92.

**Boydell, J., Bebbington, P., Bhavsar, V., Kravariti, E., van Os, J., Murray, R. M. & Dutta, R.** (2013). Unemployment, ethnicity and psychosis. *Acta Psychiatrica Scandinavica* **127**, 202-9.

**Boydell, J., van Os, J., McKenzie, K., Allardyce, J., Goel, R., McCreadie, R. G. & Murray, R. M.** (2001). Incidence of schizophrenia in ethnic minorities in London: ecological study into interactions with environment. *BMJ* **323**, 1336-8.

**Bresnahan, M., Begg, M. D., Brown, A., Schaefer, C., Sohler, N., Insel, B., Vella, L. & Susser, E.** (2007). Race and risk of schizophrenia in a US birth cohort: another example of health disparity? *International Journal of Epidemiology* **36**, 751-8.

**Brugha, T., Jenkins, R., Bebbington, P., Meltzer, H., Lewis, G. & Farrell, M.** (2004). Risk factors and the prevalence of neurosis and psychosis in ethnic groups in Great Britain. *Social Psychiatry and Psychiatric Epidemiology* **39**, 939-46.

**Bruxner, G., Burvill, P., Fazio, S. & Febbo, S.** (1997). Aspects of psychiatric admissions of migrants to hospitals in Perth, Western Australia. *Australian and New Zealand Journal of Psychiatry* **31**, 532-42.

**Cantor-Graae, E. & Pedersen, C. B.** (2007a). Risk for schizophrenia in intercountry adoptees: a Danish population-based cohort study. *Journal of Child Psychology Psychiatry* **48**, 1053-60.

**Cantor-Graae, E. & Pedersen, C. B.** (2007b). Risk of schizophrenia in second-generation immigrants: a Danish population-based cohort study. *Psychological Medicine* **37**, 485-94.

**Cantor-Graae, E. & Pedersen, C. B.** (2013). Full spectrum of psychiatric disorders related to foreign migration: a Danish population-based cohort study. *JAMA Psychiatry* **70**, 427-35.

**Cantor-Graae, E., Pedersen, C. B., McNeil, T. F. & Mortensen, P. B.** (2003). Migration as a risk factor for schizophrenia: a Danish population-based cohort study. *British Journal of Psychiatry* **182**, 117-22.

**Cantor-Graae, E. & Selten, J. P.** (2005). Schizophrenia and migration: a meta-analysis and review. *American Journal of Psychiatry* **162**, 12-24.

**Cantor-Graae, E., Zolkowska, K. & McNeil, T. F.** (2005). Increased risk of psychotic disorder among immigrants in Malmo: a 3-year first-contact study. *Psychological Medicine* **35**, 1155-63.

**Carpenter, L. & Brockington, I. F.** (1980). A study of mental illness in Asians, West Indians and Africans living in Manchester. *British Journal of Psychiatry* **137**, 201-5.

**Cochrane, R.** (1977). Mental illness in immigrants to England and Wales: an analysis of mental hospital admissions, 1971. *Social Psychiatry* **12**, 25-35.

**Coid, J. W., Kirkbride, J. B., Barker, D., Cowden, F., Stamps, R., Yang, M. & Jones, P. B.** (2008). Raised incidence rates of all psychoses among migrant groups: findings from the East London first episode psychosis study. *Archives of General Psychiatry* **65**, 1250-8.

**Dealberto, M. J., Middlebro, A. & Farrell, S.** (2011). Symptoms of schizophrenia and psychosis according to foreign birth in a Canadian sample of homeless persons. *Psychiatric Services* **62**, 1187-93.

**Gabrielsen, G. & Kramp, P.** (2009). Forensic psychiatric patients among immigrants in Denmark--diagnoses and criminality. *Nordic Journal of Psychiatry* **63**, 140-7.

**Goater, N., King, M., Cole, E., Leavey, G., Johnson-Sabine, E., Blizard, R. & Hoar, A.** (1999). Ethnicity and outcome of psychosis. *British Journal of Psychiatry* **175**, 34-42.

**Hitch, P. J. & Clegg, P.** (1980). Modes of referral of overseas immigrant and native-born first admissions to psychiatric hospital. *Soc Sci Med Med*  **14A**, 369-74.

**Hjern, A., Wicks, S. & Dalman, C.** (2004). Social adversity contributes to high morbidity in psychoses in immigrants--a national cohort study in two generations of Swedish residents. *Psychological Medicine* **34**, 1025-33.

**Hogerzeil, S. J., van Hemert, A. M., Veling, W. & Hoek, H. W.** (2017). Incidence of schizophrenia among migrants in the Netherlands: a direct comparison of first contact longitudinal register approaches. *Social Psychiatry and Psychiatric Epidemiology* **52**, 147-154.

**Hollander, A. C., Dal, H., Lewis, G., Magnusson, C., Kirkbride, J. B. & Dalman, C.** (2016). Refugee migration and risk of schizophrenia and other non-affective psychoses: cohort study of 1.3 million people in Sweden. *BMJ* **352**, i1030.

**King, M., Coker, E., Leavey, G., Hoare, A. & Johnson-Sabine, E.** (1994). Incidence of psychotic illness in London: comparison of ethnic groups. *BMJ* **309**, 1115-9.

**Kirkbride, J. B., Barker, D., Cowden, F., Stamps, R., Yang, M., Jones, P. B. & Coid, J. W.** (2008). Psychoses, ethnicity and socio-economic status. *British Journal of Psychiatry* **193**, 18-24.

**Kirkbride, J. B., Hameed, Y., Ankireddypalli, G., Ioannidis, K., Crane, C. M., Nasir, M., Kabacs, N., Metastasio, A., Jenkins, O., Espandian, A., Spyridi, S., Ralevic, D., Siddabattuni, S., Walden, B., Adeoye, A., Perez, J. & Jones, P. B.** (2017a). The Epidemiology of First-Episode Psychosis in Early Intervention in Psychosis Services: Findings From the Social Epidemiology of Psychoses in East Anglia [SEPEA] Study. *Americvan Journal of Psychiatry* **174**, 143-153.

**Kirkbride, J. B., Hameed, Y., Ioannidis, K., Ankireddypalli, G., Crane, C. M., Nasir, M., Kabacs, N., Metastasio, A., Jenkins, O., Espandian, A., Spyridi, S., Ralevic, D., Siddabattuni, S., Walden, B., Adeoye, A., Perez, J. & Jones, P. B.** (2017b). Ethnic Minority Status, Age-at-Immigration and Psychosis Risk in Rural Environments: Evidence From the SEPEA Study. *Schizophrenia Bulletin* **43**, 1251-1261.

**Kroll, J., Yusuf, A. I. & Fujiwara, K.** (2011). Psychoses, PTSD, and depression in Somali refugees in Minnesota. *Social Psychiatry and Psychiatric Epidemiology* **46**, 481-93.

**Krupinski, J. C.** (1980). Migration and Mental Health - A Comparative Study. *Journal of Intercultural Studies* **1**, 49-57.

**Leao, T. S., Sundquist, J., Frank, G., Johansson, L. M., Johansson, S. E. & Sundquist, K.** (2006). Incidence of schizophrenia or other psychoses in first- and second-generation immigrants: a national cohort study. *Journal of Nervous and Mental Disease* **194**, 27-33.

**Leao, T. S., Sundquist, J., Johansson, L. M., Johansson, S. E. & Sundquist, K.** (2005). Incidence of Mental Disorders in Second-Generation Immigrants in Sweden: A Four-Year Cohort Study *Ethnicity and Health* **10**, 243-256.

**Manhica, H., Hollander, A. C., Almquist, Y. B., Rostila, M. & Hjern, A.** (2016). Origin and schizophrenia in young refugees and inter-country adoptees from Latin America and East Africa in Sweden: a comparative study. *British Journal of Psychiatry Open* **2**, 6-9.

**McGovern, D. & Cope, R. V.** (1987). First psychiatric admission rates of first and second generation Afro Caribbeans. *Social Psychiatry* **22**, 139-49.

**McGrath, J., El-Saadi, O., Cardy, S., Chapple, B., Chant, D. & Mowry, B.** (2001). Urban birth and migrant status as risk factors for psychosis: an Australian case-control study. *Social Psychiatry and Psychiatric Epidemiology* **36**, 533-6.

**Mitter, P., Reeves, S., Romero-Rubiales, F., Bell, P., Stewart, R. & Howard, R.** (2005). Migrant status, age, gender and social isolation in very late-onset schizophrenia-like psychosis. *International Journal of Geriatric Psychiatry* **20**, 1046-51.

**Mitter, P. R., Krishnan, S., Bell, P., Stewart, R. & Howard, R. J.** (2004). The effect of ethnicity and gender on first-contact rates for schizophrenia-like psychosis in Bangladeshi, Black and White elders in Tower Hamlets, London. *International Journal of Geriatric Psychiatry* **19**, 286-90.

**Mortensen, P. B., Cantor-Graae, E. & McNeil, T. F.** (1997). Increased rates of schizophrenia among immigrants: some methodological concerns raised by Danish findings. *Psychological Medicine* **27**, 813-20.

**Nielssen, O., Sara, G., Lim, Y. & Large, M.** (2013). Country of birth and hospital treatment for psychosis in New South Wales. *Social Psychiatry and Psychiatric Epidemiology* **48**, 613-20.

**Norredam, M., Garcia-Lopez, A., Keiding, N. & Krasnik, A.** (2009). Risk of mental disorders in refugees and native Danes: a register-based retrospective cohort study. *Social Psychiatry and Psychiatric Epidemiology* **44**, 1023-9.

**Norredam, M., Garcia-Lopez, A., Keiding, N. & Krasnik, A.** (2010). Risk of mental disorders in family reunification migrants and native Danes: a register-based historically prospective cohort study. *International Journal of Public Health* **55**, 413-9.

**Qassem, T., Bebbington, P., Spiers, N., McManus, S., Jenkins, R. & Dein, S.** (2015). Prevalence of psychosis in black ethnic minorities in Britain: analysis based on three national surveys. *Social Psychiatry and Psychiatric Epidemiology* **50**, 1057-64.

**Reeves, S. J., Sauer, J., Stewart, R., Granger, A. & Howard, R. J.** (2001). Increased first-contact rates for very-late-onset schizophrenia-like psychosis in African- and Caribbean-born elders. *Britidsh Journal of Psychiatry* **179**, 172-4.

**Rwegellera, G. G.** (1977). Psychiatric morbidity among West Africans and West Indians living in London. *Psychologial Medicine* **7**, 317-29.

**Schofield, P., Thygesen, M., Das-Munshi, J., Becares, L., Cantor-Graae, E., Pedersen, C. & Agerbo, E.** (2017). Ethnic density, urbanicity and psychosis risk for migrant groups - A population cohort study. *Schizophrenia Research* **190**, 82-87.

**Schrier, A. C., van de Wetering, B. J., Mulder, P. G. & Selten, J. P.** (2001). Point prevalence of schizophrenia in immigrant groups in Rotterdam: data from outpatient facilities. *European Psychiatry* **16**, 162-6.

**Selten, J. P., Laan, W., Kupka, R., Smeets, H. M. & van Os, J.** (2012). Risk of psychiatric treatment for mood disorders and psychotic disorders among migrants and Dutch nationals in Utrecht, The Netherlands. *Social Psychiatry and Psychiatric Epidemiology* **47**, 271-8.

**Selten, J. P., Veen, N., Feller, W., Blom, J. D., Schols, D., Camoenie, W., Oolders, J., van der Velden, M., Hoek, H. W., Rivero, V. M., van der Graaf, Y. & Kahn, R.** (2001). Incidence of psychotic disorders in immigrant groups to The Netherlands. *British Journal of Psychiatry* **178**, 367-72.

**Sundquist, K., Frank, G. & Sundquist, J.** (2004). Urbanisation and incidence of psychosis and depression: follow-up study of 4.4 million women and men in Sweden. *British Journal of Psychiatry* **184**, 293-8.

**van Os, J., Castle, D. J., Takei, N., Der, G. & Murray, R. M.** (1996). Psychotic illness in ethnic minorities: clarification from the 1991 census. *Psychological Medicine* **26**, 203-8.

**Veling, W., Hoek, H. W., Selten, J. P. & Susser, E.** (2011). Age at migration and future risk of psychotic disorders among immigrants in the Netherlands: a 7-year incidence study. *American Journal of Psychiatry* **168**, 1278-85.

**Veling, W., Selten, J. P., Veen, N., Laan, W., Blom, J. D. & Hoek, H. W.** (2006). Incidence of schizophrenia among ethnic minorities in the Netherlands: a four-year first-contact study. *Schizophrenia Research* **86**, 189-93.

**Westman, J., Johansson, L. M. & Sundquist, K.** (2006). Country of birth and hospital admission rates for mental disorders: a cohort study of 4.5 million men and women in Sweden. *European Psychiatry* **21**, 307-14.

**Wijesinghe, C. P. & Clancy, D. J.** (1991). Schizophrenia in migrants living in the western region of Melbourne. *Australian and New Zealand Journal of Psychiatry* **25**, 350-7.
