# Supplementary figures and images for: Migration and psychosis: a meta-analysis of incidence studies
Source: Psychol Med. 2019 Feb 6;50(2):303–13. doi: 10.1017/S0033291719000035 (PMC7083571; doi:10.1017/S0033291719000035)

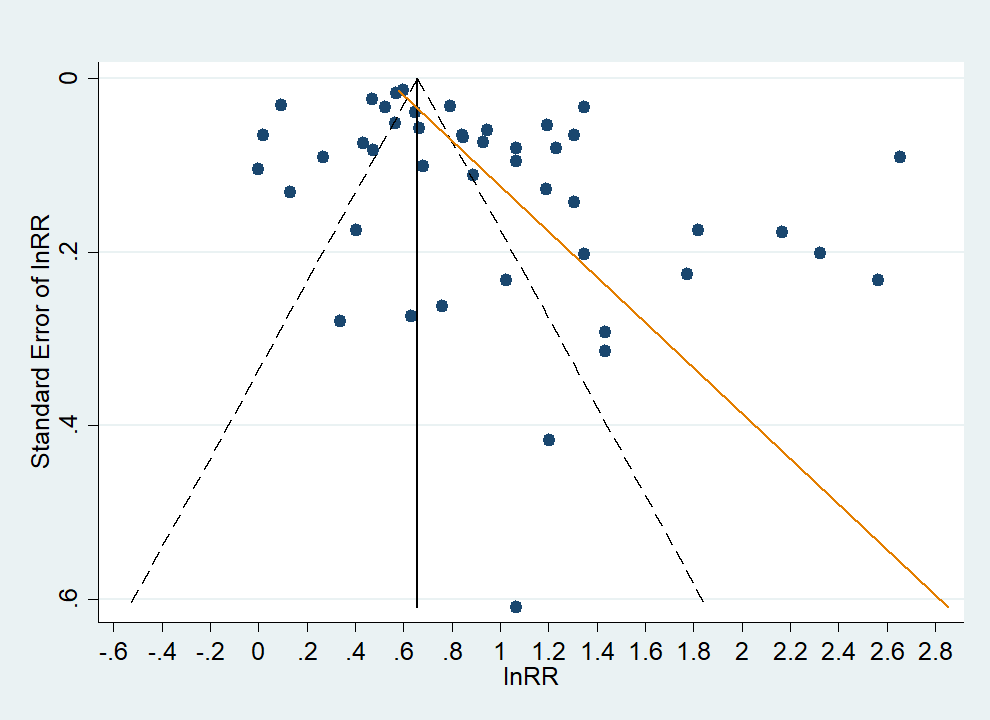

Supplement: Supplementary file 1 [file S0033291719000035sup001.zip › PsychMedSupplFig2A.PNG]

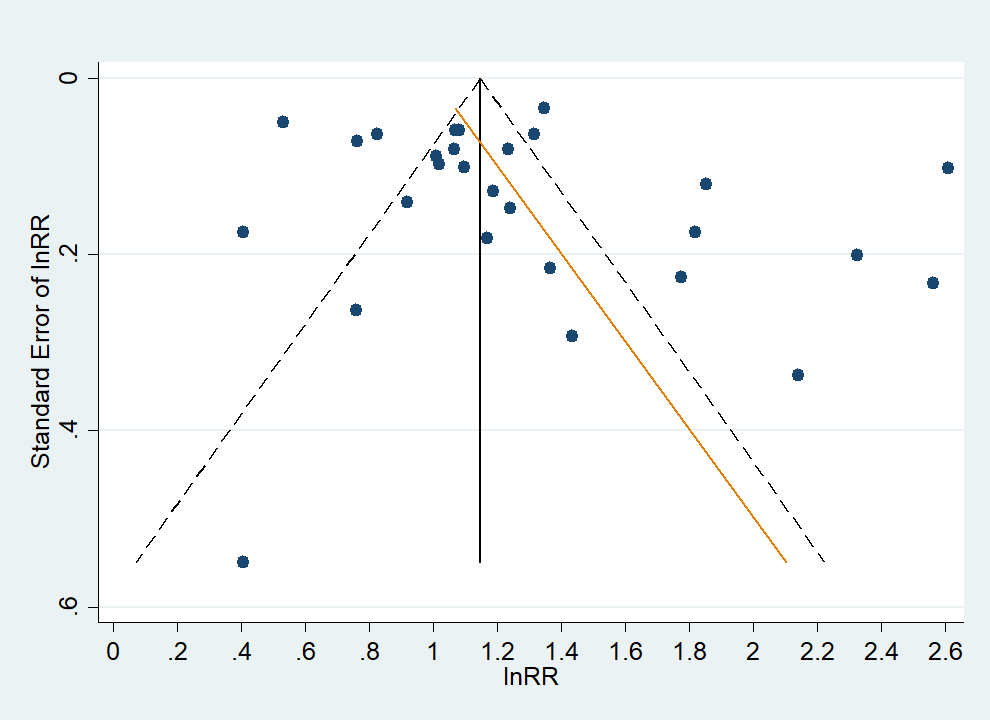

Supplement: Supplementary file 1 [file S0033291719000035sup001.zip › PsychMedSupplFig2B.PNG]
